# Dataset on infrared spectroscopy and X-ray diffraction patterns of Mg–Al layered double hydroxides by the electrocoagulation technique

**DOI:** 10.1016/j.dib.2019.104564

**Published:** 2019-09-25

**Authors:** Marena Molano-Mendoza, Dayana Donneys-Victoria, Nilson Marriaga-Cabrales, Miguel Angel Mueses, Gianluca Li Puma, Fiderman Machuca-Martínez

**Affiliations:** a Escuela de Ingeniería Química, Universidad del Valle, A.A. 25360 Cali, Colombia; b Department of Chemical Engineering, Universidad de Cartagena, A.A. 1382, Postal 195, Cartagena, Colombia; c Department of Chemical Engineering, Loughborough University, Loughborough LE11 3TU, United Kingdom

**Keywords:** *Layered double hydroxides*, *Al and AZ31 magnesium alloy electrodes*, *Electrochemical synthesis*, *Electrocoagulation*

## Abstract

The XRD profiles and FTIR analysis of sludge aggregates, *Mg–Al layered double hydroxides,* produced during electrocoagulation processes are presented. The data describes the composition of materials (LDH) produced at different operations conditions (atmospheric conditions and Mg^2+^/Al^3+^ ratio). The data show the diffraction peaks of (003), (006), (018) and (110) crystal planes for hydrotalcite structure.

**Table:** Specifications Table

Subject area	*Chemical Engineering*
More specific subject area	*Lamellar materials*
Type of data	*Table, image*, *graph, figure*
How data was acquired	*X-ray diffraction (XRD) patterns were recorded using a X'pert PRO – PANalytical diffractometer under the following conditions:* 45 kV, 40 mA*, monochromatic CuKα radiation (λ* = *0.*1542 nm*) over a in the 2θ range from of 4° to -90°. The FTIR spectra was recorded with a JASCO FT/IR-4100 over a frequency in a range of 500-4000 cm-1. The samples were prepared by mixing the powdered solids with KBr.*
Data format	*Raw data are tabulated and analyzed*
Experimental factors	*The XRD and FTIR analysis were performed according to the LDHs typical characterization*
Experimental features	*The LDH materials were prepared by electrocoagulation method with varying operations conditions and M*^*2+*^*/M*^*3+*^*ratio*
Data source location	*Universidad del Valle, Cali, Colombia*
Data accessibility	*The data are presented in this article*
Related research article	M. Molano-Mendoza, D. Donneys-Victoria, N. Marriaga-Cabrales, M. A. Mueses, G. Li Puma and F. Machuca-Martínez, Synthesis of Mg–Al layered double hydroxides by electrocoagulation, MethodsX, Volume 5, pp. 915–923, 2018.

**Table:** 

**Value of the Data** • The data set shows the methodology to obtain *Layered Double Hydroxides (LDHs)* through electrocoagulation (EC) method varying atmospheric conditions and M^2+^/M^3+^ ratio. • X-ray characterization discloses a “classical” 2H-polytype (*Magnesite)* of LDHs as well as common LDHs impurities. FTIR analysis indicates some interesting stretching and bending bonds that can have an effect on the type of material. • The EC method can guide other researchers toward designing multifunctional LDHs by using other metal electrodes (Zn, Fe, Co) for environmental applications such as water/ground remediation, solar energy storage or conversion and catalysis support.

## 1 Data

**Fig. 1:**
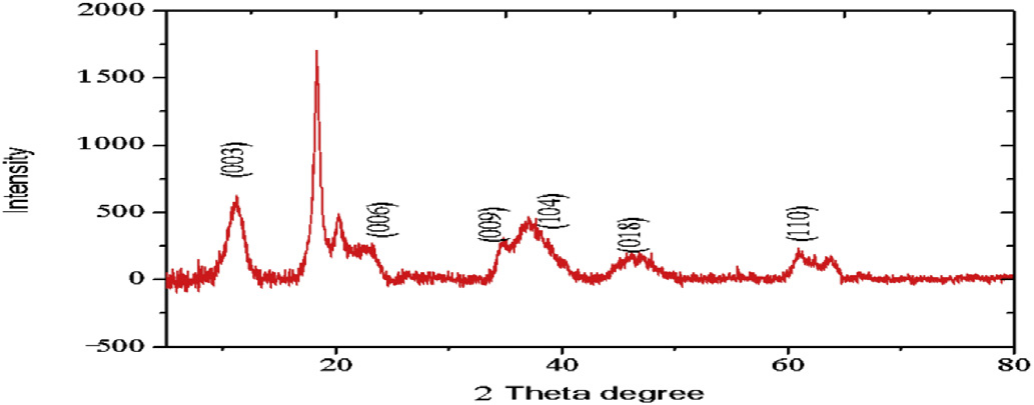
XRD pattern of the AZ31-AZ31-1 material.

**Fig. 2:**
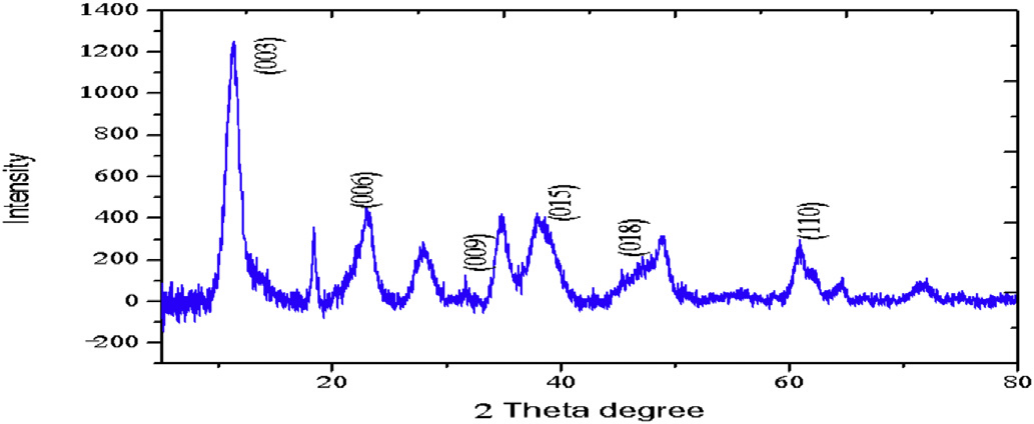
XRD pattern of the AZ31-Al-N2-1 material.

**Fig. 3:**
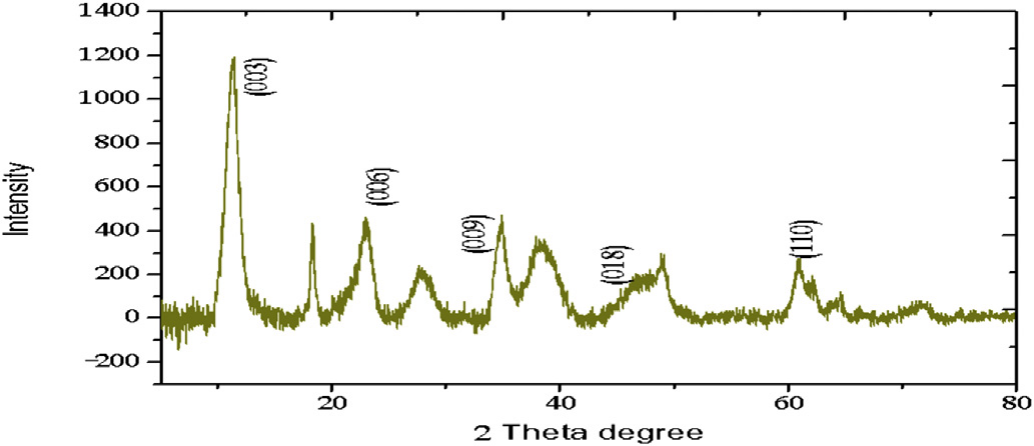
XRD pattern of the AZ31-Al-N2-3 material.

**Fig. 4:**
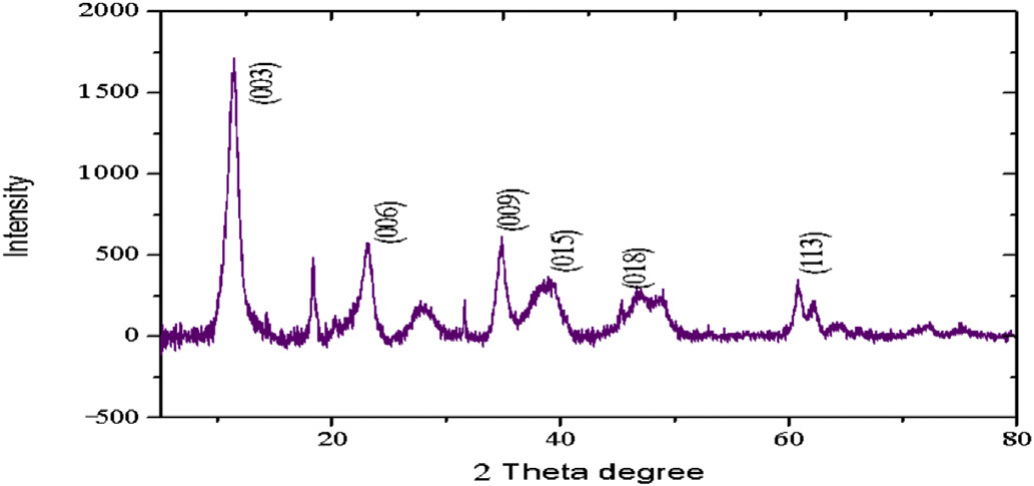
XRD pattern of the HTX3-1 material.

**Fig. 5:**
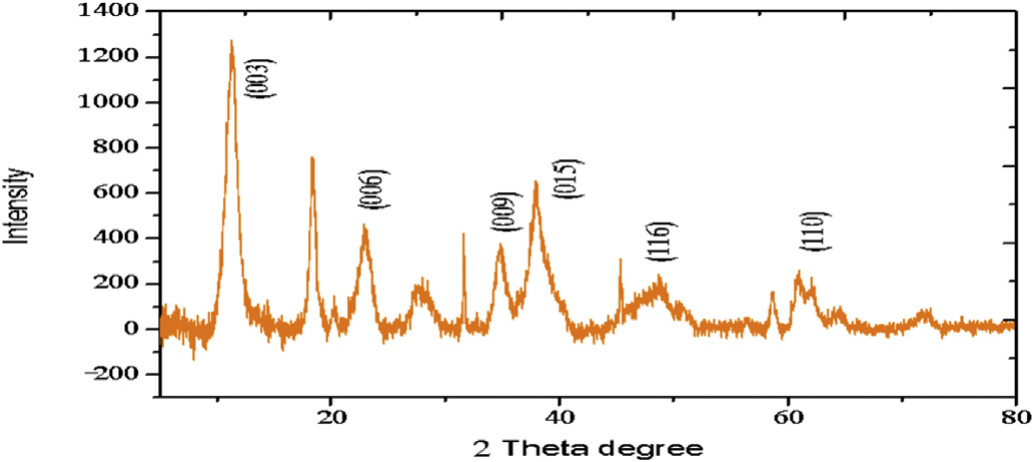
XRD pattern of the MgHP-1 material.

**Fig. 6:**
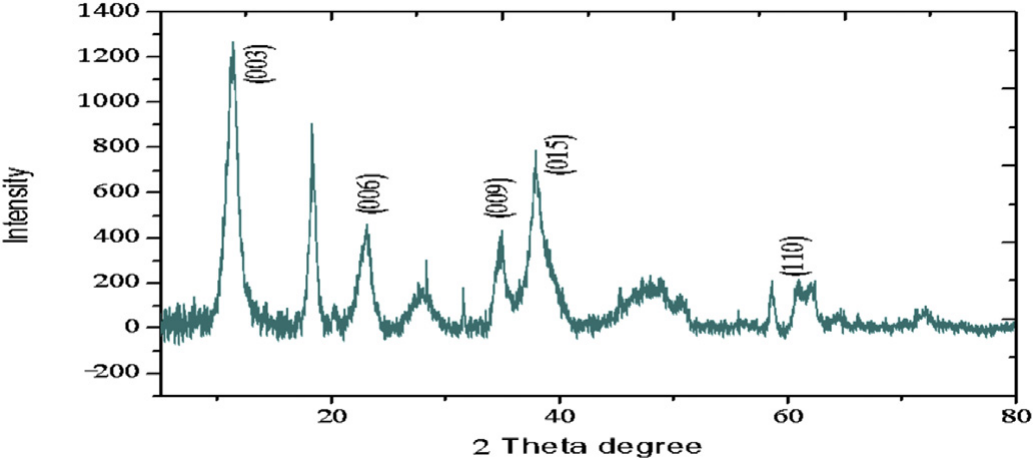
XRD pattern of the MgHP-2 material.

**Fig. 7:**
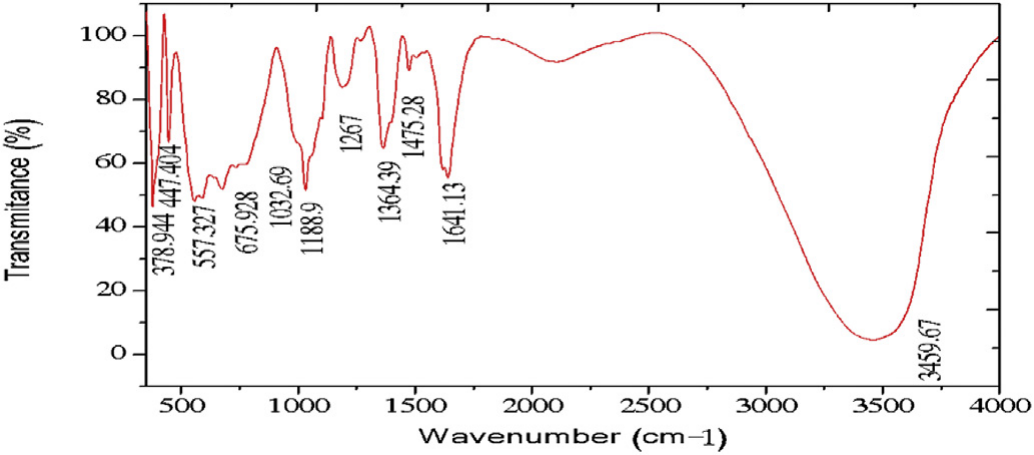
IR Spectrum of the AZ31-AZ31-1 material.

**Fig. 8:**
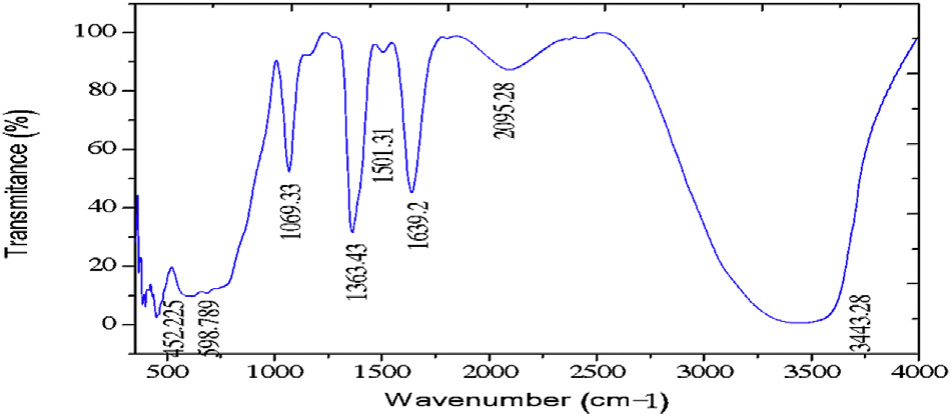
IR Spectrum of the AZ31-AL-N2-1 material.

**Fig. 9:**
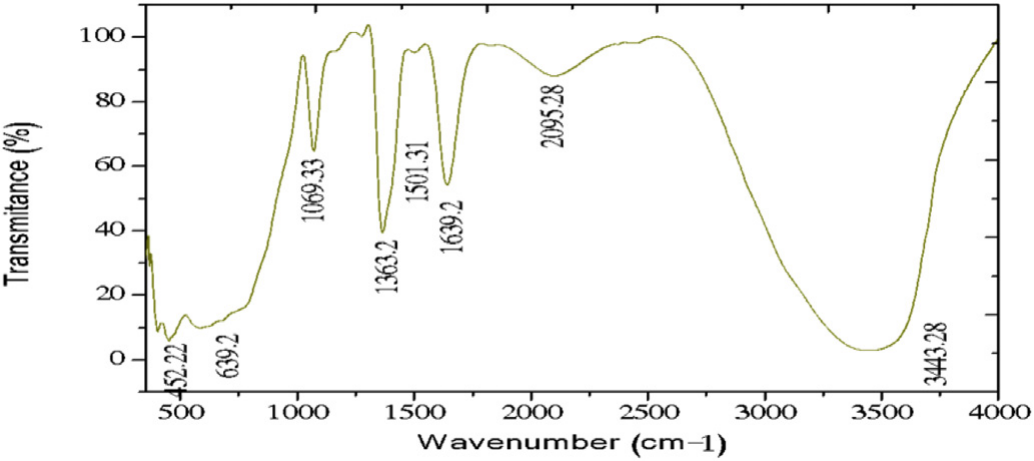
IR Spectrum of the AZ31-AL-N2-3 material.

**Fig. 10:**
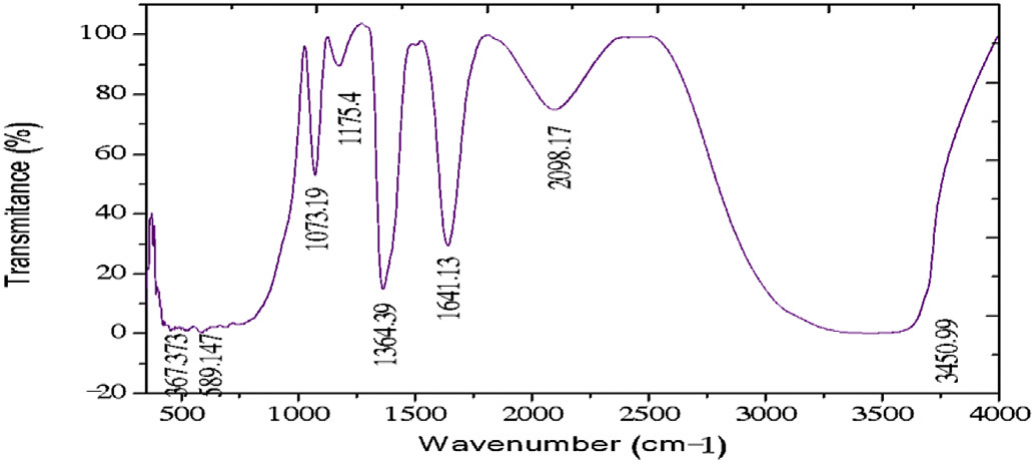
IR Spectrum of the HTX3-1 material.

**Fig. 11:**
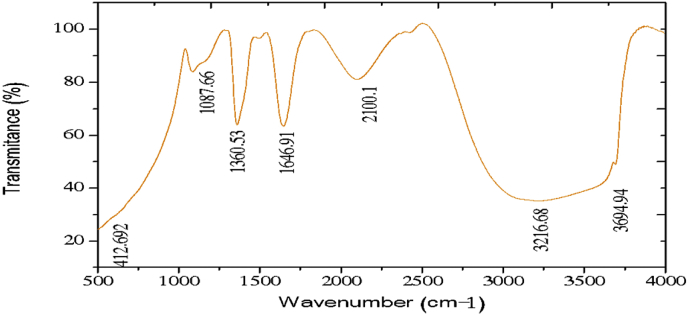
IR Spectrum of the MgHP-1 material.

**Fig. 12:**
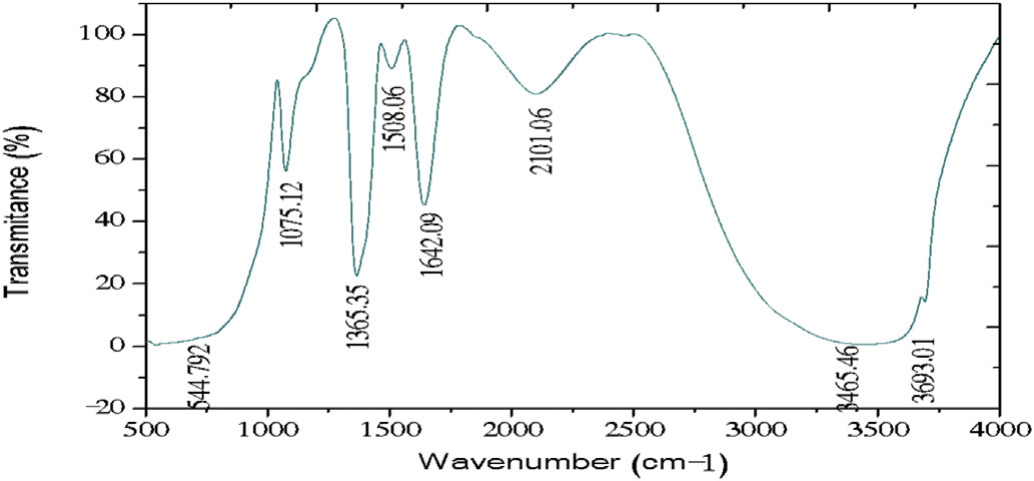
IR Spectrum of the MgHP-2 material.

The electrochemical method for the synthesis of Layered Double Hydroxides (LDHs) by electrocoagulation is used as an alternative procedure [1]. The LDHs are a class of anionic clays which have observed increasing attention due to their applications in many research areas [2]. Therefore, physicochemical properties of HDL materials, mainly explored from X-ray diffraction and FTIR analysis, disclose their more specific applications. The dataset presents LDH characteristics prepared by electrocoagulation varying atmospheric conditions and Mg^2+^/Al^3+^ ratio. Fig. 1, Fig. 2, Fig. 3, Fig. 4, Fig. 5, Fig. 6 show the diffraction peaks of (003), (006), (018) and (110) crystal planes for hydrotalcite structure. Table 1, Table 2, Table 3, Table 4, Table 5, Table 6 describe information on the phases and hkl -diffraction planes. Table 7 shows the band positions in the FTIR spectra. Fig. 7, Fig. 8, Fig. 9, Fig. 10, Fig. 11, Fig. 12 displays the functional groups and bonding information. Table 8 exhibits the LDH-material specifications.

**Table 1:** X-ray diffraction planes related to the AZ31-AZ31_(1)_MMH material.

Magnesium Aluminium Hydroxide Carbonate Hydrate (0.5%)		Hydrotalcite (0.5%)		Carbon (97.6%)		Magnesite (1.2%)		Doyleite (0.2%)	
JCPDS: 98-004-0937 Lattice parameters (Å):		JCPDS: 98-000-6183 Lattice parameters (Å):		JCPDS: 98-003-1976 Lattice parameters (Å):		JCPDS: 98-006-6643 Lattice parameters (Å):		JCPDS: 98-004-9607 Lattice parameters (Å):	
a	3.0810	a	3.054	a	14.26	a	4.314	a	4.983
b	3.0810	b	3.054	b	14.26	b	4.314	b	5.000
c	23.784	c	22.81	c	14.26	c	12.775	c	5.168
2 Theta degree	hkl	2 Theta degree	Hkl	2 Theta degree	Hkl	2 Theta degree	Hkl	2 Theta degree	Hkl
11.154	0 0 3	11.630	0 0 3	10.737	1 1 1	37.00	1 0 4	18.560	0 0 1
22.409	0 0 6	23.382	0 0 6	17.578	0 2 2	47.192	1 1 3	20.731	1 -1 0
34.419	0 1 2	34.098	1 0 1	20.643	1 1 3	61.105	1 1 6	21.263	1 0 0
36.892	1 0 4	34.792	0 1 2	21.570	2 2 2	63.276	0 1 8	21.723	0 1 0
38.657	0 1 5	35.390	0 0 9	35.583	0 4 4			22.926	0 1 -1
45.651	0 1 8	37.455	1 0 4	37.273	1 3 5			23.779	1 0 -1
45.738	0 0 12	39.343	0 1 5	37.825	0 0 6			35.526	1 1 -1
61.243	1 1 3	46.811	0 1 8	39.956	0 2 6			36.002	0 1 -2
61.393	1 0 13	60.593	1 1 0	45.382	1 1 7			37.114	1 -2 1
		60.868	0 0 15	45.850	0 4 6			37.637	0 0 2
		61.933	1 1 3	47.687	2 4 6			38.766	2 -1 -1
		63.596	1 0 13	60.893	4 6 6			46.031	1 -2 2
				62.033	1 3 9			46.242	1 -2 -1
				63.910	4 4 8			60.163	2 -2 -2
								61.920	2 0 -3
								63.865	1 -1 -3

**Table 2:** X-ray diffraction planes related to the Al-AZ31_N2 material.

Carbon dioxide (0.2%)		Hydrotalcite (0.3%)		Nitrogen oxide (0.2%)		Magnesium zinc (98.3%)		Sodium carbide (0.3%)		Magnesite (0.7%)	
JCPDS: 98-000-4494 Lattice parameters (Å):		JCPDS: 98-004-0936 Lattice parameters (Å):		JCPDS: 98-000-7431 Lattice parameters (Å):		JCPDS: 98-007-4545 Lattice parameters (Å):		JCPDS: 98-005-6296 Lattice parameters (Å):		JCPDS: 98-006-6646 Lattice parameters (Å):	
a	5.624	a	3.046	a	5.67	A	14.025	A	6.756	a	4.278
b	5.624	b	3.046	b	5.67	B	14.083	B	6.756	b	4.278
c	5.624	c	22.77	c	5.67	C	14.486	C	6.756	c	12.546
2 Theta degree	hkl	2 Theta degree	Hkl	2 Theta degree	hkl	2 Theta degree	Hkl	2 Theta degree	hkl	2 Theta degree	Hkl
27.447	1 1 1	11.646	0 0 3	27.220	1 1 1	12.210	0 0 2	22.777	1 1 1	27.947	0 1 2
35.668	0 2 1	23.421	0 0 6	31.531	0 0 2	12.562	0 2 0	37.626	0 2 2	37.540	1 0 4
39.206	1 1 2	34.194	1 0 1	35.368	0 2 1	12.611	2 0 0	61.312	0 2 4	47.716	1 1 3
48.525	1 2 2	34.882	0 1 2	38.875	1 1 2	21.674	2 2 2			62.015	1 1 6
61.657	1 2 3	35.447	0 0 9	48.105	1 2 2	23.201	1 2 3			64.469	0 1 8
		37.546	1 0 4	61.105	1 2 3	23.223	2 1 3				
		39.446	0 1 5			23.437	1 3 2				
		46.922	0 1 8			23.635	3 2 1				
		47.899	0 0 12			26.893	3 3 0				
		60.768	1 1 0			27.675	0 2 4				
		60.980	0 0 15			28.158	0 4 2				
		62.109	1 1 3			29.409	2 3 3				
		71.608	0 2 1			34.041	1 2 5				
		72.020	2 0 2			35.611	4 0 4				
		72.360	1 1 9			36.410	0 3 5				
						37.239	3 5 0				
						38.703	3 2 5				
						39.423	0 2 6				
						39.544	6 1 1				
						45.481	0 7 1				
						45.703	7 1 0				
						46.148	2 1 7				
						46.775	2 5 5				
						48.359	6 4 2

**Table 3:** X-ray diffraction planes related to the AZ31-Al-N23 material.

Hydrotalcite (20.4%)		Carbon dioxide (15.0%)		Brucite (1.1%)		Sodium Carbonate (15.4%)		Magnesite (48.1%)	
JCPDS:98-000-6183 Lattice parameters (Å):		JCPDS: 98-001-3442 Lattice parameters (Å):		JCPDS: 98-004-4736 Lattice parameters (Å):		JCPDS: 98-003-6631 Lattice parameters (Å):		JCPDS: 98-006-6646 Lattice parameters (Å):	
a	3.054	a	5.63	a	3.148	a	5.208	a	4.278
b	3.054	b	5.63	b	3.148	b	5.208	b	4.278
c	22.810	c	5.63	c	4.779	c	6.454	c	12.546
2 Theta degree	hkl	2 Theta degree	Hkl	2 Theta degree	Hkl	2 Theta degree	hkl	2 Theta degree	Hkl
11.630	0 0 3	27.414	1 1 1	18.549	0 0 1	27.619	0 0 2	27.947	0 1 2
23.382	0 0 6	35.628	0 2 1	37.614	0 0 2	34.137	0 1 2	37.540	1 0 4
34.098	1 0 1	39.160	1 1 2	37.967	0 1 1	34.413	1 1 0	47.716	1 1 3
35.3900	0 0 9	61.588	1 2 3	62.027	1 1 1	39.945	0 2 0	62.015	1 1 6
46.811	0 1 8					46.746	0 1 3	64.469	0 1 8
60.593	1 1 0					49.252	0 2 2		
60.868	0 0 15					60.936	0 1 4		
61.933	1 1 3					61.468	1 2 2		
						61.644	0 3 0

**Table 4:** X-ray diffraction planes related to the HTX3_1 material.

Hydrotalcite (12.7%)		Halite (12.5%)		Brucite (0.7%)		Gibbsite (74.1%)	
JCPDS: 98-000-6183 Lattice parameters (Å):		JCPDS: 98-011-6223 Lattice parameters (Å):		JCPDS: 98-003-4961 Lattice parameters (Å):		JCPDS: 98-008-2783 Lattice parameters (Å):	
a	3.054	a	5.653	a	3.148	a	5.052
b	3.054	b	5.653	b	3.148	b	9.495
c	22.81	c	5.653	c	4.772	c	8.686
2 Theta degree	Hkl	2 Theta degree	hkl	2 Theta degree	Hkl	2 Theta degree	Hkl
11.630	0 0 3	27.303	1 1 1	18.577	0 0 1	18.675	0 2 0
23.382	0 0 6	31.632	0 0 2	37.671	0 0 2	22.393	1 1 1
34.792	0 1 2	45.341	0 2 2	37.979	0 1 1	27.054	1 0 2
35.390	0 0 9			62.040	1 1 1	27.736	1 2 1
37.455	1 0 4					27.819	0 2 2
39.343	0 1 5					28.669	1 1 2
46.811	0 1 8					34.984	1 3 1
47.810	0 0 12					35.509	2 0 0
60.593	1 1 0					36.989	1 1 3
60.868	0 0 15					37.871	0 4 0
61.933	1 1 3					38.269	2 1 1
						39.315	0 4 1
						60.580	1 4 4
						62.015	2 5 1
						62.493	2 4 3

**Table 5:** X-ray diffraction planes related to the MgHP-1 material.

Zinc Aluminium Hydroxide Chloride Hydrate (7.6%)		Magnesite (12.3%)		Diamond (2.3%)		Sodium carbide (40.0%)		Hydrotalcite (5.2%)		Gibbsite (32.4%)	
JCPDS: 98-005-8141 Lattice parameters (Å):		JCPDS: 98-006-6646 Lattice parameters (Å):		JCPDS: 98-005-4252 Lattice parameters (Å):		JCPDS: 98-005-6291 Lattice parameters (Å):		JCPDS: 98-000-6183 Lattice parameters (Å):		JCPDS: 98-011-2963 Lattice parameters (Å):	
a	3.083	a	4.278	a	4.591	a	6.778	a	3.054	a	8.675
b	3.083	b	4.278	b	4.591	b	6.778	b	3.054	b	5.069
c	23.47	c	12.546	c	4.591	c	12.74	c	22.81	c	12.508
2 Theta degree	hkl	2 Theta degree	Hkl	2 Theta degree	hkl	2 Theta degree	Hkl	2 Theta degree	Hkl	2 Theta degree	Hkl
11.3	0 0 3	27.947	0 1 2	39.212	002	23.206	1 1 2	11.630	0 0 3	18.287	0 0 2
22.711	0 0 6	37.540	1 0 4	48.536	112	27.991	0 0 4	23.382	0 0 6	20.293	1 1 -1
34.363	0 0 9	47.716	1 1 3			36.387	1 2 3	34.792	0 1 2	22.618	1 1 -2
38.772	0 1 5	62.015	1 1 6			37.501	2 2 0	35.390	0 0 9	27.997	1 1 -3
45.920	0 1 8	64.469	0 1 8			38.766	0 2 4	37.455	1 0 4	28.091	2 1 -1
58.983	0 0 15					46.758	1 1 6	39.343	0 1 5	28.686	1 0 2
62.002	1 0 13					47.439	2 2 4	46.811	0 1 8	28.714	2 0 -4
						48.939	2 3 1	47.810	0 0 12	31.649	3 0 2
						50.697	0 2 6	60.593	1 1 0	35.159	1 1 4
						61.093	2 4 0	60.868	0 0 15	35.385	0 2 0
						61.224	2 3 5	61.933	1 1 3	35.809	3 1 3
						61.412	1 3 6	63.586	1 0 13	38.327	1 2 -2
						61.983	0 4 4			40.117	0 2 2
						64.610	0 2 8			40.249	2 1 -5
										45.440	0 2 -3
										47.175	1 0 4
										47.287	4 1 -5
										50.512	3 1 1
										58.612	2 3 -2
										60.468	4 2 -6
										64.616	6 0 -6
										72.237	1 1 -8

**Table 6:** X-ray diffraction planes related to the MgHP_Al_2 material.

Magnesium Zinc (98.5%)		Magnesium Aluminium Hydroxide Carbonate Hydrate (0.3%)		Hydrotalcite (0.3%)		Sodium Carbonate (0.9%)	
JCPDS: 98-007-4545 Lattice parameters (Å):		JCPDS: 98-004-0937 Lattice parameters (Å):		JCPDS: 98-00-61-83 Lattice parameters (Å):		JCPDS: 98-003-6621 Lattice parameters (Å):	
a	14.025	a	3.045	a	3.054	a	9.015
b	14.083	b	3.045	b	3.054	b	5.209
c	14.48	c	22.701	c	22.81	c	6.405
2 Theta degree	Hkl	2 Theta degree	hkl	2 Theta degree	Hkl	2 Theta degree	Hkl
12.210	0 0 2	11.684	0 0 3	11.630	0 0 3	23.415	2 0 -1
12.562	0 2 0	23.492	0 0 6	23.382	0 0 6	23.762	1 1 -1
17.835	2 2 0	34.205	1 0 1	34.098	1 0 1	27.897	0 0 2
23.201	1 2 3	37.580	1 0 4	34.792	0 1 2	34.408	0 2 0
23.223	2 1 3	39.486	0 1 5	35.390	0 0 9	35.464	2 0 2
23.492	3 1 2	48.058	0 0 12	37.455	1 0 4	36.557	3 1 -1
27.675	0 2 4	60.786	1 1 0	39.343	0 1 5	38.070	3 1 1
28.413	4 2 0	61.193	0 0 15	47.810	0 0 12	47.893	4 0 -2
34.741	1 5 2	62.140	1 1 3	60.593	1 1 0	50.244	2 2 2
40.633	6 2 0	72.053	2 0 2	60.868	0 0 15	55.692	0 2 -3
45.335	4 5 3			61.933	1 1 3	58.6	2 2 -3
46.523	4 6 0			72.160	1 1 9	60.730	2 2 3
47.310	1 7 2					71.203	1 3 3
48.359	6 4 2						
50.238	5 1 6

**Table 7:** Positions of the bands (in cm-1) in the IR spectra (Fig. 7, Fig. 8, Fig. 9, Fig. 10, Fig. 11, Fig. 12) [4,5].

Vibration/Assignment		Material					
		AZ31-AZ31-1	AZ31-Al-N2	AZ31AlN2-3	HTX3-1	MgHP-1	MgHP-2
Water and hydroxyl groups	*OH stretching*					3694.94	3693.01
	*Bending*	3459.67	3443.28	3443.28	3450.99	3216.68	3465.46
	*Adsorbed water*	1641.13	1639.2	1639.2	1641.13	1646.91	1642.09
Nitrogen	*N–H stretching*		2095.28	2095.28	2098.17	2100.1	2101.06
Carbonates	*C* = *O*	1475.28	1501.31	1501.31			1508.06
	*v*_*3*_*asymmetric stretching*	1364.39	1363.43	1363.2	1364.39	1360.53	1365.35
		1267					
		675.93					
	*V*_*1*_*symmetrical stretching*	1032.69	1069.33	1069.33	1073.19	1087.66	1075.12
Others	*Al–O and Mg–O deformation*	1188.9			1175.4		
	*Mg–O*			639.2			
		557.33	598.80		589.15		544.79
	*Mg–O*	447.40	452.22	452.22		412.692	
	*Mg–O*	378.94			367.37

**Table 8:** Sample specifications.

Sample	Electrodes	Current (A)	Temperature (°C)	Sodium Chloride (ppm)	Nitrogen gas	Mg^2+^/Al^3+^ ratio
AZ31-AZ31-1	AZ31-AZ31	0.51	50	5000	–	2/1
AZ31-Al-N2-1	AZ31-AZ31	0.51	50	5000	X	2/1
AZ31-Al-N2-3	AZ31-Al	0.51	50	5000	X	3/1
HTX3-1	AZ31-Al	0.51	50	5000	–	
MgAl-1	Mg–Al	0.51	50	5000	–	2/1
MgAl-2	Mg–Al	0.51	50	5000	–	2/1

### 1.1 X-ray diffraction

X-ray diffraction (XRD) patterns of the materials were measured using an X'pert PRO-PANalytical diffractometer with CuKα radiation (λ = 0.1542nm). The data were collected in the 2ʘ range of 4–90°. Determination of the phases and diffraction planes were determined using X'pert PRO-PANalytical software [3]. In every case, hydrotalcite composite was showed. Some XRD and FTIR patterns of the composites were similar to those reported in the literature for hydrotalcite materials [4].

### 1.2 Infrared spectroscopy

The FTIR analysis was carried out in the spectral range (500–4000) cm^−1^ by a Jasco FTIR-4100 spectrometer with a resolution of 4 cm^−1^. The Fig. 7, Fig. 8, Fig. 9, Fig. 10, Fig. 11, Fig. 12 represent the FTIR spectrum of composites and different vibrations attribution of the composites are represented in Table 7.

## 2 Experimental design, materials and methods

The experimental procedure is described details by Molano-Mendoza [1]. Here the protocol is provided for nitrogen experiments, giving details that were omitted from previous research article.

Electrocoagulation experiments were conducted in a batch mode, using synthetic chloride solutions as supporting electrolyte. A 5.000 mg L-1 of Sodium Chloride solution was prepared by the dissolution of Sodium Chloride (AR grade) in deionized water giving an overall final conductivity of 8.4 μsˑcm^−1^. This solution was left to dissolve for 10 min. For nitrogen experiments, the beaker was covered and stirred with a speed of 100-rpm for 3.15 h. The sample was dried in a conventional oven for 2 h at 110 °C. The dried samples were then crushed into a fine powder using a ceramic mortar/bowl.

The electrocoagulation unit consisted on two plates that worked as anodes and cathodes, AZ31 magnesium alloy, Mg or aluminum, with an immersed area of 46.6 cm^2^ each. The distance between electrodes was 5 mm, and the solution was mixing at 100 rpm using a hot magnetic plate mixer machine. Electrodes were connected to a DC power supply and the appropriate amount of the trivalent and divalent cations were carefully added to the beaker by a manual polarity inverter unit at an applied current of 0.36 and 0.15 mA. The Mg^2+^/Al^3+^ ratio and the operating time were calculated based on Faraday's law, assuming that electro-dissolution only occurs at the anode. Before testing, electrodes were subjected to dry abrasion with emery paper No. 600 and then with abrasive paper No. 1000. Afterwards, the electrodes were rinsed with distilled water for approximately 5 min to remove traces (Table 8 describes the experimental conditions).

The following units were obtained beforehand and thoroughly cleaned:

• Digital scale
• Glass beaker (size: 1000 ml)
• Magnetic hotplate stirrer
• Spatula
• Al, Mg and AZ31 alloy electrode plates
• Sodium Chloride, AR grade
• Nitrogen (N_2_) gas pipeline
• DI water
• Ceramic mortar/bowl
• Emery paper No. 600 and abrasive paper No. 1000
